# Nutritional and Phytochemical Profile of Garden-Grown *Sanguisorba*

**DOI:** 10.3390/molecules31152713

**Published:** 2026-08-04

**Authors:** Monika Kosmala, Joanna Milala, Elżbieta Karlińska, Klaudia Kita

**Affiliations:** Institute of Food Technology and Analysis, Lodz University of Technology, Stefanowskiego 2/22, 90-537 Lodz, Poland; joanna.milala@p.lodz.pl (J.M.); elzbieta.karlinska@p.lodz.pl (E.K.); klaudiakita1998@gmail.com (K.K.)

**Keywords:** *Sanguisorba*, ‘Tanna’, *Sanguisorba menziesii*, ellagitannins, total dietary fiber

## Abstract

The nutritional composition and polyphenolic profile were determined in leaf and flowering shoots of burnet species (*Sanguisorba officinalis*, *S. officinalis* ‘Tanna’, and *S. menziesii*), with plant material further separated into leaves and stems. The results demonstrated that these herbs are valuable sources of dietary fiber, as well as being rich in protein and essential minerals. Depending on the morphological part, dietary fiber content ranged from 37.8 to 67.7 g/100 g, protein content from 5.3 to 19.5 g/100 g, and ash content from 3.8 to 9.9 g/100 g. Moreover, *Sanguisorba* species are particularly abundant in polyphenols (1–11% DM), with ellagitannins such as lambertianin C and sanguiin H-6 being the predominant constituents; in selected samples, they accounted for more than 90% of the total identified polyphenols. Due to their pleasant taste and aroma, and their dietary fiber, protein, and ellagitannin content, an attempt was made to evaluate their suitability as ingredients in food products. The results indicated that leaves are the most suitable for culinary applications into dishes such as soups and smoothies, whereas stems may serve as valuable raw material for dietary fiber- and phenolic-rich preparations. In conclusion, these findings support the promotion of edible herbs that are not only nutritious, palatable, and rich in ellagitannins, but also aesthetically valuable in garden settings and beneficial for pollinators.

## 1. Introduction

*Sanguisorba* is an edible non-toxic plant considered safe for food in many parts of the world [1]. The plant exhibits diverse biological properties, including anticancer, antioxidant, antimicrobial, antiviral, anti-Alzheimer’s, antidiabetic, and anti-inflammatory activities [2,3,4]. The herb of the Rosaceae family burnet (*Sanguisorba*) is used in traditional medicine as an antibacterial, antidiarrheal, and anti-inflammatory herb for the intestines, as well as for relieving menstrual discomfort [5,6].

The herbal raw material is a herb of great burnet—*Herba Sanguisorbae*—and a great burnet rhizome *Radix Sanguisorbae*. Morphologically, the *Sanguisorba* perennial produces flower shoots during the flowering period, which are characterized by an elongated stem (70 cm) compared to leaf shoots (50 cm). At the end of the flower shoot is a flower. By nourishing the flower, the plant provides it with many nutrients. Flowers appear only on flowering shoots, and the leaves are characterized by an elongated shape compared to the leaves on leafy shoots.

*Sanguisorba* herbs are also characterized by an attractive appearance and are a true ornament of the garden. Great burnet is a perennial with a strong thick rhizome, rubella leaves, and odd-stemmed stems up to 1 m high. Burnet blooms from June to August with purple flowers [5]. The flowers are attractive to pollinators. *Sanguisorba officinalis* is only partly self-compatible and completely dependent on the activity of pollen vectors to set seeds. The flowers are abundantly visited by a diverse assemblage of short-tongued insects attracted by pollen and nectar [7]. The most commonly occurring *Sanguisorba species* are the *Sanguisorba minor* Scop. (small burnet) and *Sanguisorba officinalis* L. (great burnet) [6,8].

*Sanguisorba officinalis* ‘Tanna’ is a perennial that attracts attention to its original, bulbous flower heads of a striking dark burgundy color. The plant forms loose, upright, airy clumps that reach about 50–60 cm in height. It has very decorative, pinnate, basal leaves, set on long stalks, which are composed of small, oval leaflets with serrated edges. *Sanguisorba officinalis* ‘Tanna’ blooms profusely from July to September, producing slender, straight, branched stems topped with charming, burgundy flower heads of an oval, elongated shape. The small, incredibly striking, fluffy pom-poms impress with their unconventional structure and intense colors and provide a true paradise for pollinating insects. *Sanguisorba officinalis* ‘Tanna’ is an undemanding, easy-to-grow plant, fully frost-resistant, which also has medicinal properties and can be used in the kitchen. It makes extraordinary decorations for perennial flower beds, wildflower meadows, and naturalistic and rustic gardens.

*Sanguisorba menziesii* is a large decorative plant with flower stalks reaching 180 cm. It is an interesting perennial but still undervalued. Thanks to its dense, dark red inflorescence set on tall stems, it looks very original. *Sanguisorba menziesii* has a loose growth habit, which is why its impressive and striking inflorescences stand out well against the foliage.

*Sanguisorba* plants consist of several morphologically distinct organs, including stems, leaves, flowers, and roots. While the young leaves are primarily used for culinary purposes, these plant parts differ considerably in both their morphology and phytochemical composition. The highest total polyphenol content was reported in the flowers, reaching 14,444.97 mg/100 g DM, with tannins accounting for 66.4% of total phenolics, whereas the lowest concentration was found in the stems (4606.33 mg/100 g DM; 43.3% tannins) [3]. Individual morphological parts also exhibited distinct biological activities. Leaf extracts showed the strongest antioxidant potential, with antiradical and reducing capacities of 6.63 and 0.30 mmol TE/g DM, respectively. In contrast, flower extracts demonstrated the greatest inhibitory effects against α-amylase and α-glucoamylase, whereas leaf extracts most effectively inhibited pancreatic lipase activity. Furthermore, extracts obtained from leaves and flowers displayed the strongest antiproliferative activity against pancreatic ductal adenocarcinoma, colorectal adenocarcinoma, bladder cancer, and T-cell leukemia cell lines [3].

Extracts from great burnet herbs showed antioxidant activity and high antimicrobial activity in a wide spectrum of test strains [6,8]. Moreover, the extracts from medicinal great burnet *Sanguisorba officinalis* strongly inhibit the growth of melanoma cells [9]. Extracts from dried roots of *Sanguisorba officinalis* exhibited anti-inflammatory activity, and histopathological studies showed that this extract inhibits the infiltration of inflammatory cells and excessive mucus secretion. These results indicate that *Sanguisorba officinalis* has therapeutic potential against bronchial asthma associated with allergic diseases [10]. In addition to their health-promoting effects, *Sanguisorba* herbs exhibit a cucumber-like aroma with a slightly nutty note and can be an interesting ingredient in dishes such as soups, sauces, and salads, as well as for flavoring alcoholic beverages [11]. To enhance the taste, this plant was added to cheese, butter, ice cream drinks, fresh orange juice, and kiwi juice with vinegar. Fresh leaves from young plants are used as a seasoning for salads and meat dishes in some Western countries. In China, *Sanguisorba officinalis* is considered an important tonic food and is often added to dishes such as porridge or nettle soup [12].

The numerous bioactive compounds are responsible for the indicated biological properties of plants of the genus *Sanguisorba* spp. Over 120 compounds including phenols, flavonoids, neolignans, and terpenoids have been isolated from the genus *Sanguisorba* so far [2]. The most important polyphenolic components found in the discussed plants include tannins, mainly: ellagitannins and flavonols. The roots of *Sanguisorba officinalis* contain tannins in the amount of 12–17% [6]. The herbs of *Sanguisorba officinalis* contain 14.5 phenolics, in these 13% are ellagitannins, then flavonols 0.8%, free ellagic acid 0.6%, and hydroxycinnamic acids 0.1% [4]. While tannins in food products are divided into hydrolysable (ellagitannins, ET) and condensed tannins (proanthocyanidins, PC), the *Sanguisorba officinalis* is especially rich in ellagitannins [4]. Ellagitannins exhibit antioxidant, anti-inflammatory effects [13], lower blood cholesterol levels, and positively affect the gut microbiome [14,15,16,17]. Ellagitannins, esters of ß-D-glucose and hexahydroxy diphenic acid (HHDP), form a diverse group comprising both simple and complex molecules. In the group there are simple ETs, C-glycosidic ETs, complex tannins (condensates of C-glycosidic tannins with flavan 3-ol), and oligomers up to pentamers [18]. Large molecule ellagitannins do not enter the bloodstream. In the large intestine, under the influence of the microbiome, ellagitannins undergo hydrolysis to urolithins, with ellagic acid and monomeric ellagitannins being more easily metabolized to urolithins than dimeric ellagitannins [19]. Urolithins show antioxidant, anti-inflammatory, and anti-estrogen/estrogen activity [20] and are detected in body fluids such as blood or urine [21,22].

Procyanidins are structurally diverse compounds that can be classified as monomers, oligomers, and polymers depending on the degree of polymerization. Since these low molecular weight procyanidins (degrees of polymerization less than 3) are completely absorbed in the gastrointestinal tract and relatively easy to purify, most research is focused on oligomers. They exhibit broad benefits to human health and are used in the prevention of cancers, cardiovascular diseases, and diabetes [23]. Hydroxycinnamic acids, like chlorogenic acid, are found in a wide range of plants and as they combine antioxidant, anti-inflammatory, and metabolic functions, which can be utilized for therapeutic purposes in humans [24]. Quercetin and its glucosides express the antioxidant potency as well as the ability to inhibit angiotensin-converting enzyme (ACE) activity, acetylcholinesterase (AChE) activity, and advanced glycation end product (AGE) formation showing their importance against hypertension, Alzheimer-type dementia, and diabetic complication, respectively [25]. The aim of the study was to assess the nutritional and polyphenol content of *Sanguisorba* herbs (*S. officinalis*, *S. officinalis* ‘Tanna,’ and *S. menziesii*), which are decorative and worth planting in the garden, especially because, in addition to their interesting appearance, they are attractive to pollinating insects. The three *Sanguisorba* species (*S. officinalis*, *S. officinalis* ‘Tanna’, and *S. menziesii*) were selected because they are attractive ornamental plants suitable for garden cultivation and differ markedly in their morphology and growth habit. The comparison of leaves, stems, flowering shoots, and leafy shoots was undertaken because these morphological parts differ substantially in appearance and physiological function. Flowering shoots are typically longer and bear smaller leaves than vegetative leafy shoots, reflecting different growth and developmental stages. These morphological differences may be associated with variations in nutritional composition and phytochemical profiles, which constituted the primary focus of the present study.

The division used in the study into leaf shoots and flower shoots was intended to answer the question about differences in qualitative and quantitative composition between the intensively growing elongated flower shoot and the shorter leaf shoot. Moreover, the plant shows strong morphological differences between the stem and the leaves. Usually, the herbal raw material consists of leaves. The stems are hard and often fibrous, so only young stems can be used for salads and cooked dishes. Dishes with great burnet were also prepared—three smoothies and three soups—and subsequently subjected to sensory evaluation.

## 2. Results

### 2.1. Nutritional Content

All samples contained high dry matter levels (93.3–95.8 g/100 g) (Table 1). The highest concentration of dry matter was found in the stems. In most cases, the standard deviation did not exceed 0.5 g/100 g.

The ash content serves as an indicator of the overall number of mineral components in plant material. The lowest value was found in the flower shoot stems of *S. menziesii*, where the ash content was 3.8 g/100 g. The highest level was recorded in the stems of *S. officinalis* ‘Tanna’ flower shoot stems reaching 9.9 g/100 g. The fat content in the plant analyzed raw materials was relatively low but showed clear differences between leaves and stems. The highest lipid concentrations were found in the leafy shoot leaves of *S. menziesii* (3.8 g/100 g), *S. officinalis* leafy shoot leaves (3.7 g/100 g), as well as in the leaves of the same plant in flower shoot leaves (3.5 g/100 g).

Protein, as a key nutrient, was present in significantly higher amounts in the leaves than in the stems. The highest content of this compound was recorded in the leaves of *S. officinalis* flower shoot leaves, where it reached 19.5 g/100 g. Conversely, the lowest protein concentration was found in the leafy shoot stems of the same plant—5.3 g/100 g. In the case of *S. menziesii*, the protein content in the leaves ranged from 15.6 g/100 g to 17.1 g/100 g, while in the stems it was 6.1–6.4 g/100 g. The ‘Tanna’ variety deserves special attention, in which the protein content in the leaves—both those with flower shoots and those without—was remarkably similar and amounted to 15.8 g/100 g.

The level of dietary fiber in the examined plant material was high, particularly in the case of stems. It was precisely these parts of the plant that were characterized by the highest fiber concentration. The highest values were recorded in the leafy shoot stems of *S. officinalis* of the ‘Tanna variety’—67.7 g/100 g, as well as in the flower shoot stems of *S. officinalis*—67.1 g/100 g. In case of *S. menziesii*, the fiber content in the stems ranged from 57.6 to 66.3 g/100 g. The leaves contained smaller amounts of fiber, although they still constituted a significant component of these tissues, from 37.8 to 46.8 g/100 g. The stems of *S. menziesii* exhibited significant fiber levels whereas the leaves of *S. officinalis* exhibited the highest phenolic content. The content of metabolizable carbohydrates was calculated as the difference between 100 and the sum of protein, fat, water, total ash, dietary fiber, and phenolics content. Phenolics were calculated as a sum of ellagitannins, chlorogenic and neochlorogenic acids, ellagic acid and its derivatives, flavonols (UHPLC-PDA-MS), and flavan-3-ols (phloroglucinolysis). The highest concentrations of metabolized carbohydrates were found in the leafy shoot leaves of the ‘Tanna’ variety, where they reached 23.8 g/100 g. The lowest carbohydrate content was recorded in the *S. officinalis* flower shoot stems (11.5 g/100 g) and in the leafy shoot stems of the ‘Tanna’ variety (11.1 g/100 g). Leaves contained more metabolized carbohydrates than stems. The largest difference between flowering and vegetative leafy shoots was observed in the phenolic content. Leaves of flowering shoots of *S. officinalis* contained significantly more phenolics (11.5 g/100 g DM) than leaves of vegetative shoots (6.5 g/100 g DM). No comparable differences were found in *S. officinalis* ‘Tanna’ or *S. menziesii*, indicating that shoot developmental stage affected phenolic accumulation only in *S. officinalis*.

### 2.2. Phenolics

**Figure 1:**
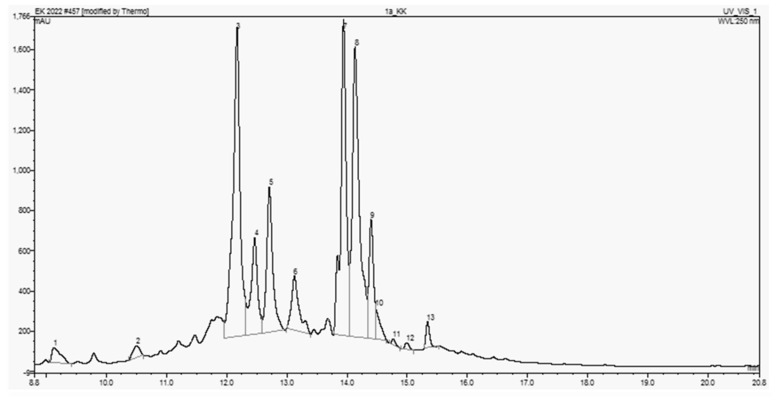
*Sanguisorba* officinalis chromatogram. 1. neochlorogenic acid, 2. chlorogenic acid, 3. lambertianin C isomer, 4. sanguiin H6, 5. lambertianin C isomer, 6. galloyl-bis-HHDP glucose isomer, 7. lambertianin C isomer, 8. ellagic acid, 9. quercetin glucoside, 10. quercetin glucuronide, 11. ellagic acid pentoside isomer, 12. ellagic acid pentoside isomer, 13. quercetin rhamnoside.

Table 2 presents detailed results of the determination of the content of individual polyphenolic compounds in the leaves of great burnet collected at the flowering stage. Table 3 and Figure 1 present the phenolics identification. The content of procyanidins in *Sanguisorba officinalis*, its variety ‘Tanna’, and *Sanguisorba menziesii* is presented in Table 4. In Table 1 the phenolics are presented as a sum of HPLC determination (Table 2) and flavonol-3-ols determination by phloroglucinolysis (Table 4). The most abundant phenolic content was found in *S. officinalis* flower shoot leaves (11,474 mg/100 g). In case of leafy shoot leaves, the content of phenolics was lower by half. In the stems, the phenolics content was much lower 449–644 mg/100 g. In case of *S. menziesii* the highest number of phenolics was found in leafy shoot leaves, 6418 mg/100 g, while in the stems there was 1917 mg/100 g of phenolics. Flower shoot leaves and flower shoot stems in the case of *S. menziesii* had similar content of phenolics, 2342 and 2531, respectively. In the case of *S. officinalis* ‘Tanna’, the content of phenolics was similar in the leaves (2510–2755 mg/100 g) and in the stems (976–1248 mg/100 g), no matter if it had a leafy shoot or a flower shoot.

Procyanidins were present in all samples, while free catechins were completely absent (Table 4). The procyanidin content was highly variable and ranged widely from 9.5 mg/100 g to as much as 535.8 mg/100 g. The highest concentration of these compounds was found in the flower shoot leaves and in the flower shoot stems of *S. menziesii*, 535.8 and 274.3 mg/100 g, respectively, while in leafy shoot leaves procyanidins were only 28.1 and in leafy shoot stems 124.4 mg/100 g. In *S. officinalis* ‘Tanna’ in the leaves there was 120–126 mg/100 g of procyanidins and 21–25.6 mg/100 g in the stems. In *S. officinalis*, the content of procyanidins was the lowest; in the leaves there was 20–22.2 mg/100 g and 9.5–13.5 mg/100 g in the stems.

Among the compounds (Table 3 and Table 4), ellagitannins predominated, especially lambertianin C isomers, then sanguiin H6, galloyl-bis-HHDP-glucose isomer, and ellagic acid pentoxides and free ellagic acid. Flavonols, mainly quercetin glycosides, were also present, along with minor amounts of hydroxycinnamic acids such as chlorogenic and neochlorogenic acid.

### 2.3. Sensory Evaluation of Food Products

The assessment of product sensory attributes on a points scale is not sufficient for manufacturers. It does not provide an answer as to why the product received such a rating and what needs to be done to improve its quality. Therefore, during sensory evaluation, a ranking assessment is also used. During this evaluation, the consumer rates each of the sensory attributes on a points scale, indicating what they believe an ideal product should possess.

The sensory evaluation of the prepared dishes was conducted with a group of 10 people. The rating ranged from 1 to 10, with 10 being the highest possible score. Table 5 presents the average scores of the prepared smoothie variants, while Table 6 shows the average scores of the prepared soups.

From the averages of the sensory attribute scores of the prepared smoothie variants collected in Table 5, compared to the product identified as ideal by the evaluators, smoothie variant 3 performed the best. The second-best product in terms of consumer preference was smoothie 1. According to the average consumer ratings, smoothie 2 differed the most from the preferred ideal product, with only the color attribute being close to the ideal.

From the averages of the sensory attribute scores of the prepared soup variants collected in Table 6, compared to the product identified as ideal by the evaluators, soup 4 performed the best. According to the evaluators, soup 5 turned out to be the second-best variant that appealed to tastes. The soup that was least like the ideal was soup 6.

The sensory evaluation was conducted with a relatively small panel (*n* = 10), which limits the generalizability of the findings and does not allow firm conclusions regarding broader consumer acceptance.

The sensory assessment involved untrained volunteers and was intended as a preliminary evaluation of the acceptability of soups and smoothies enriched with *Sanguisorba* species. As the study did not aim to perform a comprehensive consumer analysis, detailed demographic stratification was not included.

## 3. Discussion

The aerial parts of herbs *Sanguisorba* were found to be a source of polyphenols, especially ellagitannins, with a higher quantity compared to other sources of these polyphenols such as berries. In raspberries the total content of polyphenols was 127 to 160 mg/100 g FW, with DM 13–15% [27], which corresponds to 846 and 1231 mg/100 g DW.

Similar results regarding the high polyphenol content in different morphological parts of *Sanguisorba officinalis* were reported by Lachowicz et al. [3]. The authors found that the total phenolic content reached 14,445, 9963, 8687, and 4606 mg/100 g DM in flowers, leaves, roots, and stalks, respectively. Using LC-DAD-ESI-QTOF-MS/MS analysis, they identified 130 polyphenolic compounds, including 62 compounds reported in *S. officinalis* for the first time. Tannins were the predominant group of phenolics, accounting for 66.4% of total polyphenols in flowers and 43.3% in stalks. These findings are consistent with the present study, confirming that *Sanguisorba* morphological parts differ considerably in both polyphenolic composition and the accumulation of bioactive compounds.

Ansari et al. [1] found that *Sanguisorba minor* hydroalcoholic extract is no-toxic in research on mice and rats. Heidari et al. [2] reviewed that *Sanguisorba* spp. the presence of phenolic compounds, flavonoids, and terpenoids, which were responsible for anticancer, antioxidative, antimicrobial, antiviral, anti-Alzheimer’s disease, and anti-inflammatory activity.

Finimundy et al. [8] identified 22 phenolics in *S. minor*, including 5 phenolic acids, 7 flavonoids, and 10 tannins. The predominant phenolics detected in leaves were sanguiin H-10, lambertianin C, and pedunculagin (bis-HHDP-glucoside), then galloyl-HHDP-glucoside and quercetin-*O*-glucuronide. In roots, (+)-catechin and B-type (epi)catechin dimer and tetramer were found in the majority. In general, roots contained higher amounts of total phenolic acids, total hydrolysable tannins, and total flavonoids than leaves. In leaves there was 105–122 mg/g of total phenolics and in the roots 165–298 mg/g, respectively. In the samples examined, the leaves of *Sanguisorba officinalis* reached a similar order of magnitude in polyphenol content. Furthermore, we found that the leaves contain more polyphenols than the stems. Flowering shoots in the case of S. officinalis contained more polyphenols than leaf shoots. In the case of *S. menziesii* it was the opposite, and in *S. officinalis* ‘Tanna’ the values were similar.

Lachowicz et al. [3] using the sensitive LC-DAD-ESI-QTOF-MS/MS technique found the presence of 130 polyphenols in *S. officinalis* L., 62 compounds were detected in for the first time. Tannins were the dominant group of phenolics, with contents ranging from 66.4% of total polyphenols in the flowers to 43.3% in the stalks. The highest content of polyphenols was identified in the flowers and reached 14,444.97 mg/100 g, while the lowest was noted in the stalks and reached 4606.33 mg/100 g. The extracts expressed in vitro antioxidant activity, the ability of inhibition of α-amylase, α-glucoamylase, and pancreatic lipase activity. Moreover, the leaves and flowers showed the most effective antiproliferative properties in pancreatic ductal adenocarcinoma, colorectal adenocarcinoma, bladder cancer, and T-cell leukemia cells. In the stalks the activity was the weakest.

Finimundy et al. [8] found that *S. minor* extracts expressed antimicrobial activities, especially against *Salmonella typhimurium*. Moreover, root samples revealed activity against all tested cell lines (four tumor cell lines, namely HeLa (cervical carcinoma), HepG2 (hepato cellular carcinoma), MCF-7 (breast adenocarcinoma), NCI-H460 (non-small cell lung cancer), and non-tumor PLP2 (porcine liver cells). All the root samples showed toxic effects against the tested non-tumor PLP2 cell line (at 231 mg/mL), while leaves were effective only against HeLa cell line and non-toxic to normal cell lines. Also, Tan et al. [9] found that *Sanguisorba officinalis* extracts strongly inhibit the growth of B16F10 melanoma cells and identified ellagic acid as the responsible ingredient. Gawron-Gzella et al. [6] found that *S. officinalis* herb extracts expressed antioxidant activity as well as antimicrobial activity on five Gram-positive bacteria (*S. aureus*, *S. epidermidis*, *M. luteus*, *B. subtilis*, *E. faecalis*), three Gram-negative bacteria (*E. coli*, *K. pneumoniae*, *P. aeruginosa*), and fungi (*C. albicans*).

Tocai (Moţoc) et al. [28] found the ethanol extract obtained from the leaves of *S. minor* Scop. contains the highest content of phenolic compounds at 160.96 mg/g dw compared to the flower and root extract (131.56 mg/g dw and 121.36 mg/g dw, respectively). In *S. officinalis*, they found the highest number of phenols in the root extract (127.06 mg/g) compared to the flower and leaves extract (102.31 mg/g and 81.09 mg/g dw, respectively). The ethanol extract of *S. minor* Scop. leaves exhibited the best antibacterial activity against *Staphylococcus aureus*, *Escherichia coli*, and *Pseudomonas aeruginosa*.

Karlińska et al. [29] found that in a three-year-old agrimony herb, a herb from the Roseacea family, from a field crop the polyphenolic content of *Agrimony procera* was 3.9%, 3.2%, 2.9%, 1.8% and 1.1%, and that of *A. eupatoria* was determined to be 1.3%, 0.3%, 0.9%, 0.6% and 0.5% in the dry matter of leaves, stems, fruits, seeds and hypanthia, respectively. Leaves of *S. menzienzii* and *S. officinalis* ‘Tanna’ are comparable with leaves of *A. procera*, the herb from the same family as *Sanguisorba*. And similarly leaves are a better source of polyphenols than stems as in agrimony. In case of *A. procera* cultivated with additional nitrogen dose the content of polyphenols reached 10, 8, 12, 18% in stems, roots, underground buds, respectively with flavon-3-ols content 3–4%, 2–3%, 3–4, and 2% in stems, roots, underground buds, respectively [30]. In our previous research, they found that the edible medicinal plants *Sanguisorba officinalis* L. (great burnet), *Geum urbanum* L. (wood avens), and *Agrimonia procera* Wallr. (fragrant agrimony) of the Rosaceae family contained 3.0, 2.1, and 3.4 g/100 g phenolics in fresh matter [4].

In *Sanguisorba* herbs, the content of flavan-3-ols is relatively low. In *S. officinalis*, up to 22 mg/100 g, *S. menziesii* was the richest in procyanidins from the investigated herbs, up to 535.8 mg/100 g.

Moreover, *Sanguisorba* herbs are a rich source of dietary fiber. Our previous research also showed that aerial parts of herbs are a thorough source of dietary fiber (6.5 for *Sanguisorba*, 8.2 for *Geum*, and 11.1 g/100 g fresh matter for *Agrimonia*) and vitamin C, which are all 0.1 g/100 g of fresh matter [4]. Moreover, due to their resistance to fungal diseases and pathogens, the medicinal plants are free from pesticide residues. In the present study, stems contained substantially higher levels of dietary fiber than leaves. Although stems are generally less suitable for direct culinary use because of their texture, their high fiber content, together with the presence of phenolic compounds, suggests considerable potential for valorization as functional food ingredients. These fractions could be utilized in the development of fiber-rich preparations, food supplements, or functional food products aimed at increasing dietary fiber intake while providing additional phytochemicals. Therefore, stems should not be considered merely a by-product but rather a valuable plant material with potential applications in the food industry.

The plant materials examined were found to contain significant amounts of ash, which may indicate that the studied herbs are a source of minerals. The mineral composition should be examined in further studies.

It can be stated that leaves are a better source of polyphenols and protein but contain less dietary fiber. Karlińska et al. [30] studying *A. procera*, also found greater amounts of dietary fiber, smaller amounts of protein, and smaller amounts of minerals in the stems compared to the leaves. However, higher amounts of polyphenols were found in the leaves compared to the stems. Nevertheless, due to the phloem, it is likely that the leaves would rather be consumed and stems would rather be used for hot beverages or phenolic extracts.

Fresh leaves of *Sanguisorba* were used to produce tasty smoothies and soups proving in consumer analysis that they are acceptable. In Western countries, *Sanguisorba officinalis* is primarily valued as a culinary herb. Fresh young leaves are used as a seasoning for salads and meat dishes, contributing to mild, refreshing, cucumber flavor. This positions the plant mainly as a flavor-enhancing culinary ingredient [2]. In medieval European cuisine, *S. minor* was often used as an addition to hot one-pot dishes, as well as to flavor alcoholic beverages. Finely chopped herbs are suitable for seasoning herb butter and cottage cheese; they go well with white cream sauce for fish, and fillings for chicken and pork. *S. officinalis* is less aromatic than *S. minor*, but also edible [11]. In China, the plant plays a dual culinary and medicinal role. It is considered an important tonic food, reflecting its place in traditional dietary therapy. *Sanguisorba officinalis* is commonly added to porridge or nettle soup, demonstrating its integration into warm, restorative dishes typically associated with health benefits [12].

The *Sanguisorba* herbs are attractive for pollinators in the garden. The flowers of *S. minor* and *S. officinalis* were both regularly visited by insects that encountered the reproductive organs and might thus act as pollinators. The species were visited by various species of beetles, flies, and bees, but the proportions of the diverse groups varied depending on the locality. While the flower visitors of *S. minor* are only attracted by pollen as nectar was not present in the flowers of this species, the flowers of *S. officinalis* produce pollen but also small amounts of nectar being more attractive to the flower visitors [7].

## 4. Materials and Methods

### 4.1. Plant Materials

The plant material was collected at the end of June. The material was obtained from a cultivation located near Lodz. During harvest, the plants were in the flowering stage. Harvesting was done by hand, cutting the stems with shears at a height of about 15 cm (10 cm) above the ground. The plant stems were placed in plastic bags and immediately transported to the Institute of Food Technology and Analysis at the Lodz University of Technology for separation into morphological parts. Three independent biological replicates were used for each species (ca. 1.5 kg from each plant, *S. officinalis*, *S. officinalis* ‘Tanna’ *S. menziesii*). For *S. officinalis* ‘Tanna’ three plants (length 45 cm) had a combined weight of 1.8 kg, including 9% of flowering shoots. The shoots of *S. menziesii* reached a length of 180 cm, with leaves constituting 21% of the total mass, 38% stems, and shoots with flowers 41%. In *S. officinalis*, the leaf shoots measured 50 cm in length, whereas the flower shoots reached 70 cm, with leaves constituting 21% of the total mass, stems 38%, and shoots with flowers 41%. Leaf shoots are shorter than flower shoots. Flowers appear only on flower shoots, and the leaves are characterized by an elongated shape compared to the leaves on leaf shoots. Leaf shoots were separated from flower shoots and then divided into leaves and stems. The material was naturally air-dried in the shade.

### 4.2. Methods

AOAC [31]: Protein content of fruit products—AOAC 920.152; fat (Ether Extract of Plants) AOAC 930.09; dry matter—and ash content of fruit—AOAC 940.26, total dietary fiber in foods (TDF) AOAC 985.29. Extraction of polyphenols: two step extraction; first with 70% methanol with 0.1% HCOOH, then with 70% acetone with 0.1% HCOOH. Each repeated 3 times at room temperature with sonification for 15 min. Sample 0.5 g, 3 mL of solvent added 3 times, filled up to 10 mL. Extracts combined. UHPLC analysis: Dionex Ultimate 3000 UHPLC with DAD and MS detection (Q Exactive Orbitrap, Thermo Fisher Scientific, Waltham, MA, USA). Column Luna Omega 1.6 µm C18 100 Å (150 × 2.1 mm) (Phenomenex, Torrance, CA, USA), temperature 40 °C, detection at 250 nm, 280 nm, 320 nm, and 360 nm, flow 0.4 mL/min. Gradient: 0–2 min, 5% phase B; 2–12 min, 5–28% phase B; 12–20 min, 28–73% phase B; 20–25 min, 73%; 25–27 min, 73–5% phase B; and 27–30 min 5% phase B. Phenolic compounds were quantified based on the corresponding calibration curves. Ellagitannins were quantified as agrimoniin equivalents at 250 nm, chlorogenic acid and neochlorogenic acid at 320 nm, and flavonols, ellagic acid, and ellagic acid derivatives at 360 nm. Ellagic acid (2.55–25.5 mg/L, R^2^ = 1, LOD = 0.31 mg/L, LOQ = 0.93 mg/L), agrimoniin (ET, obtained previously, 5.75–217.01 mg/L, R^2^ = 0.999, LOD = 0.18 mg/L, LOQ = 0.54 mg/L), chlorogenic acid (2.06–20.6 mg/L, R^2^ = 0.999, LOD = 1.05 mg/L, LOQ = 3.19 mg/L), quercetin-3-*O*-glucoside (5.10–51.0 mg/L, R^2^ = 1, LOD = 0.35 mg/L, LOQ = 1.05 mg/L), and quercetin-3-*O*-rhamnoside (9.35–93.5 mg/L, R^2^ = 0.999, LOD = 0.58 mg/L, LOQ = 1.19 mg/L) (Extrasynthese, Genay, France) were used as standards. The mass spectrometer recorded spectra in negative mode (H-ESI source). The source parameters were vaporizer temperature, 500 °C, ion spray voltage, 4 kV, and capillary temperature, 400 °C; the sheath gas and auxiliary gas flow rates were 75 and 20 units, respectively. In full MS/dd-MS2 scanning mode, the mass range was set at *m*/*z* 200–2000 and the collision energy at 20 eV. Xcalibur software 3.063 (Thermo Fisher Scientific, Waltham, MA, USA) was used. Phenolic compounds were calculated based on the prepared calibration curves of standards, ellagitannins were calculated at a wavelength of 250 nm, and phenolic acid, flavonols, and flavones at a wavelength of 360 nm.

Flavan-3-ols were determined by phloroglucinolysis [32]. Briefly, 0.8 mL of solution containing 75 g/L phloroglucinol and 15 g/L ascorbic acid in methanol was added to 20 mg of sample; then 0.4 mL of 0.2 mol/L HCl in methanol was added to start phloroglucinolysis lasting 30 min at 50 °C. The reaction was stopped in an ice bath by adding 0.6 mL of 0.04 mol/L sodium acetate. The adducts were injected into HPLC with a fluorescence detector RF-10AXL (Shimadzu, Tokyo, Japan), and a Gemini 5u C18 110a (250 4.60 mm; 5 m) column (Phenomenex, Torrance, CA, USA). The following gradient was used: 0–10 min, 4–7% phase B; 10–27 min, 7–30% phase B; 27–29 min, 30–70% phase B; 29–34 min, 70% phase B; 34–35 min, 70–4% phase B; and 35–40 min, 4% phase B. Phase A was 2.5% acetic acid in water, and phase B was 80% acetonitrile in water. The flow was 1 mL/min, the column temperature was 25 °C. The standards were used: (−)-epicatechin, (+)-catechin, (−)-epicatechin-phloroglucinol and (+)-catechin-phloroglucinol. The FD was set at a 278 nm excitation wavelength and a 360 nm emission wavelength. (−)-epicatechin-phloroglucinol adduct R^2^ = 0.9974 LOD mg/L 0.34 LOQ mg/L 0.68, (−)-epicatechin R^2^ = 0.9999 LOD mg/L 0.03 LOQ mg/L, 0.06, and (+)-catechin R^2^ = 0.9999 LOD mg/L 0.04 LOQ mg/L 0.084.

### 4.3. Statistics

Three independent biological replicates were used for each species and analyzed in duplicate. Means, standard deviations, and statistical analyses are based on biological replicates. The test results were subjected to a statistical analysis using the one-way analysis of variance with Duncan’s post hoc test and was performed at a significance level of 0.05 using the Statistica 10 software (Stat Soft, Tulsa, OK, USA). Assumptions of normality and homogeneity of variances were verified before analysis.

### 4.4. Herb Dishes and Their Sensory Evaluation

The dishes were prepared using fresh leaves of *Sanguisorba officinalis*.

#### 4.4.1. Smoothies

Three smoothies were prepared. All the ingredients of each smoothie variant were placed in a blender jug and then finely chopped, bringing the ingredients together. Smoothie 1 was prepared from: water 250 g, 151 g of apple flesh, 107 g of peeled banana, 38 g of *Sanguisorba* leaves, 21 g of frozen raspberries, and 18 g of peeled lemon. Smoothie 2 was prepared from: water 250 g, 122 g of frozen raspberries, 42 g of celery stalk, 38 g of *Sanguisorba* leaves, 7 g of peeled ginger, and 0.3 g of cinnamon. Smoothie 3 was prepared from: water 200 g, 111 g of peeled banana, 39 g of *Sanguisorba* leaves, 38 g of peeled cucumber, 22 g of peeled lemon, and 4 g of mint leaves.

#### 4.4.2. Soups

The developed soup variants were prepared as follows. All ingredients for each soup variant were placed in a pot and heated over a burner until all vegetables became tender. After cooking, the soups were cooled, and the vegetable pieces present in the broth were blended using a hand blender. All soup variants were prepared using the same procedure. Soup 4 was prepared from: 1000 g of water, 315 g of peeled potato, 75 g of peeled carrot, 58 g of great burnet (*S. officinalis*), 9 g of peeled garlic, 1 g of salt, and 0.5 g of pepper. Soup 5 was prepared from: 500 g of water, 197 g of tomato, 59 g of great burnet, 49 g of peeled carrot, 56 g of celery, 8 g of peeled garlic, 1 g of salt, and 0.7 g of pepper. Soup 6 was prepared from: 500 g of water, 242 g of peeled potato, 237 g of tomato, 49 g of great burnet, 1.5 g of salt, and 0.6 g of pepper.

#### 4.4.3. Sensory Analysis

A sensory evaluation of the prepared dishes was conducted with a group of 10 people. It must be considered the preliminary sensory assessment as only ten untrained participants were involved. During the evaluation of the prepared soups, saltiness and spiciness were also assessed. In addition to evaluating the dishes themselves, consumers also indicated what values of individual attributes they thought an ideal product should have. The rating ranged from 1 to 10, with 10 being the highest possible score. From the obtained ratings, averages for each attribute were calculated for each variant of the prepared dishes. The sensory evaluation was conducted in accordance with institutional guidelines, and participation was voluntary. The sensory assessment involved untrained volunteers.

## 5. Conclusions

*Sanguisorba* herbs are exceptionally rich in ellagitannins. They feature a pleasant taste and aroma. These plants are also highly recommended for garden cultivation due to their decorative red flowers, which attract pollinators—an important group of organisms that deserve active protection. Therefore, it is worthwhile promoting herbs that are edible, tasty, contain ellagitannins, and at the same time are ornamental and beneficial for pollinators. Among the examined species, *Sanguisorba officinalis* contains the highest number of polyphenols; however, *S. menziesii* and *S. officinalis* ‘Tanna’ are also rich sources of ellagitannins. Within the aerial parts, the leaves are the most suitable for culinary use, whereas the stems may serve as valuable raw material for dietary fiber and phenolic preparations. Ellagitannins and their gut microbiota-derived metabolites, urolithins, have been associated with several beneficial effects, including anti-inflammatory activity. However, the assessment of biological activities was beyond the scope of the present study. Future research should address this aspect to provide a more comprehensive evaluation of the beneficial potential of *Sanguisorba* species. Practical culinary applications of *Sanguisorba* species also deserve further attention. While the present study demonstrates the nutritional value and rich bioactive compound content of *Sanguisorba* species and includes a preliminary evaluation of soups and smoothies enriched with these herbs, further research is needed to more comprehensively assess their sensory attributes and consumer acceptance. Such studies would help determine whether *Sanguisorba* species are not only rich in ellagitannins but also attractive and palatable ingredients for a wider range of culinary applications.

## Figures and Tables

**Table 1:** Nutritional composition of Sanguisorba herbs, g/100 g.

	Dry Matter	Ash	Fat	Protein	TDF	Carbohydrates	Phenolics
*S. officinalis* FSL	93.5 ± 0.0 ^ef^	7.0 ± 0.1 ^f^	3.5 ± 0.1 ^b^	19.5 ± 0.2 ^a^	37.8 ± 3.2 ^e^	14.2 ± 3.6 ^de^	11.5 ± 0.3 ^a^
*S. officinalis* FSS	95.0 ± 0.4 ^bc^	8.2 ± 0.6 ^de^	0.8 ± 0.0 ^g^	6.7 ± 0.2 ^c^	67.1 ± 0.5 ^a^	11.5 ± 0.1 ^e^	0.7 ± 0.0 ^ef^
*S. officinalis* LSL	93.3 ± 0.0 ^f^	8.9 ± 0.1 ^bc^	3.7 ± 0.3 ^ab^	17.5 ± 0.7 ^b^	42.1 ± 1.3 ^e^	14.6 ± 2.5 ^cde^	6.5 ± 0.2 ^b^
*S. officinalis* LSS	95.1 ± 0.1 ^b^	9.4 ± 0.0 ^ab^	0.7 ± 0.0 ^g^	5.3 ± 0.4 ^c^	63.6 ± 1.5 ^ab^	15.6 ± 1.9 ^bcde^	0.5 ± 0.0 ^f^
*S. menziesii* FSL	94.1 ± 0.3 ^d^	6.8 ± 0.0 ^f^	1.6 ± 0.0 ^e^	17.1 ± 0.1 ^b^	46.8 ± 1.0 ^d^	18.9 ± 1.1 ^abcd^	2.9 ± 0.1 ^c^
*S. menziesii* FSS	95.8 ± 0.2 ^a^	3.8 ± 0.1 ^g^	1.1 ± 0.0 ^f^	6.4 ± 0.3 ^c^	66.3 ± 0.7 ^ab^	15.4 ± 0.3 ^bcde^	2.8 ± 0.1 ^d^
*S. menziesii* LSL	94.4 ± 0.1 ^cd^	7.1 ± 0.0 ^f^	3.8 ± 0.1 ^a^	15.6 ± 3.1 ^b^	41.4 ± 2.8 ^e^	20.3 ± 0.6 ^ab^	6.5 ± 0.3 ^b^
*S. menziesii* LSS	94.9 ± 0.3 ^bc^	8.1 ± 0.4 ^de^	1.7± 0.0 ^e^	6.1 ± 0.4 ^c^	57.6 ± 0.0 ^c^	19.4 ± 0.5 ^abc^	2.0 ± 0.3 ^d^
*S. officinalis* ‘Tanna’ FSL	94.1 ± 0.2 ^d^	8.7 ± 0.1 ^bcd^	2.1 ± 0.1 ^d^	15.8 ± 0.1 ^b^	42.5 ± 0.0 ^de^	22.4 ± 0.5 ^a^	2.6 ± 0.0 ^c^
*S. officinalis* ‘Tanna’ FSS	95.3 ± 0.5 ^ab^	9.9 ± 0.2 ^a^	1.1 ± 0.0 ^f^	7.1 ± 0.0 ^c^	61.8 ± 1.7 ^bc^	14.4 ± 2.0 ^cde^	1.0 ± 0.0 ^e^
*S. officinalis* ‘Tanna’ LSL	94.0 ± 0.2 ^de^	8.6 ± 0.0 ^cde^	2.4 ± 0.0 ^c^	15.8 ± 0.1 ^b^	40.6 ± 4.7 ^e^	23.8 ± 4.7 ^a^	2.8 ± 0.5 ^c^
*S. officinalis* ‘Tanna’ LSS	95.0 ± 0.3 ^a^	8.0 ± 0.7 ^e^	1.1 ± 0.0 ^f^	5.9 ± 0.1 ^c^	67.7 ± 0.0 ^a^	11.1 ± 0.3 ^e^	1.2 ± 0.0 ^ef^
*p*	0.000	0.000	0.000	0.000	0.000	0.001	0.000

The values in the columns marked with different letters differ statistically significantly at *p* ≤ 0.05, flower shoot leaves—FSL, flower shoot stems—FSS, leafy shoot leaves—LSL, leafy shoot stems—LSS.

**Table 2:** Phenolics of *Sanguisorba* herbs, mg/100 g DM.

	*S. officinalis* FSL	*S. officinalis* FSS	*S. officinalis* LSL	*S. officinalis* LSS	*S. menziesii* FSL	*S. menziesii* FSS	*S. menziesii* LSL	*S. menziesii* LSS	*S. officinalis* ‘Tanna’ FSL	*S. officinalis* ‘Tanna’ FSS	*S. officinalis* ‘Tanna’ LSL	*S. officinalis* ‘Tanna’ LSS
Neochlorogenic acid	6.4 ± 0.4 ^e^	0.1 ± 0.0 ^f^	0.1 ± 0.0 ^f^	0.1 ± 0.0 ^f^	0.1 ± 0.0 ^f^	0.1 ± 0.0 ^f^	44.8 ± 2.2 ^a^	23.5 ± 0.5 ^d^	34.5 ± 0.9 ^b^	9.2 ± 0.1 ^e^	30.2 ± 0.0 ^c^	6.1 ± 0.1 ^e^
Chlorogenic acid	28.9 ± 0.5 ^a^	1.9 ± 0.2 ^e^	26.3 ± 0.7 ^b^	2.8 ± 0.1 ^d^	1.7 ± 0.2 ^e^	0.4 ± 0.0 ^f^	27.0 ± 0.3 ^b^	0.1 ± 0.0 ^f^	0.8 ± 0.1 ^f^	0.1 ± 0.0 ^f^	12.6 ± 5.3 ^c^	1.9 ± 0.1 ^e^
Lamb.C isomer	4369.8 ± 134.7 ^a^	133.2 ± 9.0 ^d^	1433.8 ± 8.5 ^c^	18.0 ± 0.4 ^d^	121.2 ± 0.8 ^d^	119.2 ± 11.6 ^d^	3331.6 ± 718.2 ^b^	786.0 ± 105.6 ^cd^	1413.9 ± 260.6 ^c^	73.6 ± 13.9 ^d^	1240.5 ± 61.9 ^d^	53.7 ± 3.6 ^d^
S.H-6	1196.8 ± 104.5 ^a^	85.7 ± 4.2 ^f^	520.1 ± 2.6 ^d^	148.2 ± 3.7 ^f^	188.3 ± 6.0 ^f^	204.2 ±15.5 ^f^	463.8 ± 86.6 d^e^	927.8 ± 128.6 ^b^	368.7 ± 86.5 ^e^	754.6 ± 37.0 ^c^	778.2 ± 9.1 ^c^	1049.5 ± 11.7 ^b^
Lamb.C isomer	1697.1 ± 58.3 ^a^	95.1 ± 17.5 ^c^	1111.4 ± 50.4 ^b^	55.4 ± 1.3 ^cd^	2.5 ± 0.0 ^d^	11.2 ± 5.0 ^d^	34.0 ± 6.1 ^d^	97.8 ± 14.9 ^c^	23.7 ± 1.8 ^d^	15.1 ± 1.8 ^d^	27.6 ± 3.8 ^d^	26.8 ± 4.3 ^d^
Galloyl-bis-HHDP glucose isomer	520.1 ± 92.8 ^a^	46.2 ± 61.8 ^cd^	104.2 ± 2.6 ^bc^	78.2 ± 11.0 ^bcd^	2.5 ± 0.0 ^d^	5.3 ± 0.8 ^d^	151.0 ± 17.1 ^b^	2.5 ± 0.0 ^d^	6.7 ± 0.6 ^d^	10.2 ± 1.1 ^d^	14.7 ± 1.6 ^d^	2.5 ± 0.0 ^d^
Lamb.C isomer	2963.1 ± 42.7 ^a^	6.8 ± 6.0 ^d^	2184.7 ± 123.2 ^b^	2.5 ± 0.0 ^d^	1661.7 ± 42.9 ^c^	2089.1 ± 89.9 ^b^	2.5 ± 0.0 ^d^	2.5 ± 0.0 ^d^	2.5 ± 0.0 ^d^	2.5 ± 0.0 ^d^	2.5 ± 0.0 ^d^	2.5 ± 0.0 ^d^
EA	437.6 ± 2.2 ^a^	226.7 ± 4.4 ^c^	372.9 ± 15.7 ^b^	85.7 ± 2.3 ^d^	94.3 ± 37.6 ^d^	89.2 ± 5.0 ^d^	99.7 ± 0.4 ^d^	34.9 ± 2.9 ^ef^	28.3 ± 0.8 ^ef^	20.3 ±14.5 ^f^	33.4 ± 0.2 ^ef^	57.3 ± 0.7 ^e^
EA pentoside isomer	10.1 ±0.7 ^de^	26.5 ± 0.4 ^c^	6.4 ± 0.1 ^ef^	10.9 ± 5.0 ^d^	2.7 ± 0.0 ^f^	2.7 ± 0.0 ^f^	2.7 ± 0.0 ^f^	7.1 ± 0.9 ^e^	51.0 ± 2.0 ^b^	7.2 ± 0.3 ^de^	57.4 ± 1.1 ^a^	2.7 ± 0.0 ^f^
EA pentoside isomer	2.7 ± 0.0 ^e^	13.0 ± 2.6 ^c^	11.6 ± 0.3 ^c^	7.1 ± 0.2 ^d^	2.7 ± 0.0 ^e^	2.7 ± 0.0 ^e^	2.7 ± 0.0 ^e^	3.2 ± 0.7 ^e^	22.8 ± 1.0 ^b^	27.2 ± 0.0 ^a^	2.7 ± 0.0 ^e^	16.5 ± 0.4 ^e^
Quercetin glucosidee	0.3 ± 0.0 ^d^	0.3 ± 0.0 ^d^	2.9 ± 0.3 ^d^	0.3 ± 0.0 ^d^	0.3 ± 0.0 ^d^	0.3 ± 0.0 ^d^	0.3 ± 0.0 ^d^	2.0 ± 0.2 ^d^	263.1 ± 6.8 ^a^	15.7 ± 1.8 ^c^	234.2 ± 1.9 ^b^	0.3 ± 0.0 ^d^
Quercetin glucuronide	120.4 ± 5.4 ^c^	0.0 ± 0.0 ^f^	570.4 ± 20.9 ^a^	36.1 ± 32.6 ^d^	252.8 ± 7.5 ^b^	6.4 ± 2.9 ^d^	25.8 ± 0.2 ^def^	29.2 ± 3.0 ^de^	29.2 ± 0.7 ^de^	6.0 ± 0.1 ^ef^	30.1 ± 0.3 ^de^	4.6 ± 1.2 ^ef^
Quercetin rhamnoside	120.8 ± 1.6 ^c^	8.9 ± 0.2 ^g^	87.8 ± 3.3 ^d^	4.6 ± 0.6 ^gh^	11.1 ± 2.7 ^g^	0.3 ± 0.0 ^h^	0.3 ± 0.0 ^h^	0.9 ± 0.9 ^h^	265.0 ± 9.3 ^b^	35.2± 0.0 ^e^	291.0 ± 5.3 ^a^	23.2 ± 1.4 ^f^
SUM	11,474.1 ± 307.3 ^a^	644.4 ± 22.3 ^ef^	6432.6.4 ± 235.5 ^b^	449.9 ± 17.1 ^f^	2341.9 ± 104.6 ^c^	2531.1 ± 95.1 ^d^	6418.2 ± 830.8 ^b^	1917.5 ± 275.9 ^d^	2510.2 ± 506.8 ^c^	976.9 ± 30.1 ^ef^	2755.1 ± 33.2 ^c^	1247.6 ± 1.9 ^e^
*p*	0.000	0.000	0.000	0.000	0.000	0.000	0.000	0.000	0.000	0.000	0.000	0.000

Values in rows marked with different letters differ statistically significantly at *p* ≤ 0.05, flower shoot leaves—FSL, flower shoot stems—FSS, leafy shoot leaves—LSL, leafy shoot stems—LSS, Lamb.C—lambertianin C, S.H-6—sanguiin H-6, EA—ellagic acid.

**Table 3:** Phenolics of *Sanguisorba* herbs identification.

	Group	Tentative Name	RT	MS/MS, *m*/*z*	[M−H]^−^	Ref.
1	Hydroxycinnamic acid	Neochlorogenic acid	9.1	191.06	353.09	[4]
2	Hydroxycinnamic acid	Chlorogenic acid	10.8	191.06	353.09	[standard, [4]]
3	Ellagitannin	Lambertianin c isomer	12.4	783.06, 934.06; 1401.59	1869.45 z = 3	[8,26]
4	Ellagitannin	Sanguiin H6	12.7	309.06; 934.06	1870.13 z = 1	[3,27]
5	Ellagitannin	Lambertianin c isomer	12.9	783.06; 934.06; 1567.13	1869.13 z = 1	[8,26]
6	Ellagitannin	Galloyl-bis-HHDP glucose isomer	13.3	783,06; 433.04; 301.06	935.07 z = 1	[8,26]
7	Ellagitannin	Lambertianin c isomer	14.1	783.06; 934.06; 1567.13	1869.13 z = 1	[8,26]
8	Ellagic acid	Ellagic acid	14.2		301.06	[standard, [8]]
9	Flavonol	Quercetin glucoside	14.4	301.04	463.03	[standard, [4]]
10	Flavonol	Quercetin glucuronide	14.5	301.04	477.06	[8]
11	Ellagic acid derivative	Ellagic acid pentoside isomer	15.1	301.06	433.04	[8]
12	Ellagic acid derivative	Ellagic acid pentoside isomer	15.5	301.06	433.04	[8]
13	Flavonol	Quercetin rhamnoside	15.8	301.05	447.09	[standard, [4]]

Ref.—references.

**Table 4:** Flavan-3-ols of *Sanguisorba* herbs, mg/100 g DM.

	Flavan-3-ols [mg/100 g]
*S. officinalis* flower shoot leaves	22.2 ± 6.3 ^de^
*S. officinalis* flower shoot stems	9.5 ± 0.2 ^f^
*S. officinalis* leafy shoot leaves	20.1 ± 0.3 ^de^
*S. officinalis* leafy shoot stems	13.5 ± 1.8 ^ef^
*S. menziesii* flower shoot leaves	535.8 ± 4.6 ^a^
*S. menziesii* flower shoot stems	274.3 ± 4.7 ^b^
*S. menziesii* leafy shoot leaves	28.1 ± 5.2 ^d^
*S. menziesii* leafy shoot stems	124.4 ± 1.4 ^c^
*S. officinalis* ‘Tanna’ flower shoot leaves	126.3 ± 8.6 ^c^
S. officinalis ‘Tanna’ flower shoot stems	21.0 ± 0.3 ^de^
*S. officinalis* ‘Tanna’ leafy shoot leaves	120.3 ± 6.5 ^c^
*S. officinalis* ‘Tanna’ leafy shoot stems	25.6 ± 0.2 ^d^
*p*	0.000

The values in the columns marked with different letters differ statistically significantly at *p* ≤ 0.05.

**Table 5:** Sensory analysis of smoothies.

Sensory Differentiator	Smoothie 1	Smoothie 2	Smoothie 3	*p*	Perfect Product
Sour	3.0 ± 1.0 ^b^	9.0 ± 1.0 ^a^	3.0 ± 0.9 ^b^	0.000	4
Astringent	2.2 ± 1.2 ^c^	7.0 ± 1.3 ^a^	4.9 ± 1.5 ^b^	0.000	4
Bitter	2.0 ± 0.8 ^c^	7.0 ± 1.1 ^a^	3.2 ± 1.0 ^b^	0.000	2
Sweet	5.0 ± 1.0 ^a^	2.1 ± 0.7 ^c^	4.0 ± 1.1 ^b^	0.000	5
Fleshiness	3.0 ± 0.8 ^b^	2.8 ± 0.9 ^b^	4.1 ± 1.2 ^a^	0.009	5
Color	3.0 ± 0.6 ^c^	5.1 ± 1.1 ^a^	3.9 ± 1.2 ^b^	0.002	6
Smell	7.0 ± 1.3	7.9 ± 1.2	4.9 ± 1.5	0.055	5
Lumpiness	10.0 ± 0.0 ^a^	9.8 ± 0.0 ^b^	10.0 ± 0.0 ^a^	0.035	1
Consistency	1.0 ± 1.1 ^c^	2.2 ± 0.6 ^b^	4.1 ± 1.1 ^a^	0.000	10

The values in the rows marked with different letters differ statistically significantly at *p* ≤ 0.05.

**Table 6:** Sensory analysis of soups.

Sensory Differentiator	Soup 4	Soup 5	Soup 6	*p*	Perfect Product
Sour	5.0 ± 1.2 ^b^	8.0 ± 1.0 ^a^	3.0 ± 1.4 ^c^	0.000	5
Astringent	1.9 ± 0.9 ^b^	3.9 ± 1.1 ^a^	1.9 ± 0.7 ^b^	0.000	2
Bitter	2.1 ± 0.5	2.1 ± 0.8	1.9 ± 0.5	0.753	2
Sweet	1.2 ± 0.4 ^b^	2.0 ± 0.5 ^a^	1.1 ± 0.3 ^b^	0.000	2
Fleshiness	9.0 ± 1.0 ^a^	9.2 ± 1.0 ^a^	5.8 ± 0.6 ^b^	0.000	9
Color	3.8 ± 0.6	4.0 ± 1.0	3.1 ± 1.1	0.072	5
Smell	5.1 ± 0.7 ^a^	3.1 ± 1.1 ^b^	5.0 ± 1.0 ^a^	0.000	5
Lumpiness	10.0 ± 1.0	10.0 ± 0.0	10.0 ± 0.0	0.380	5
Consistency	4.9 ± 1.4 ^a^	5.1 ± 0.9 ^a^	2.8 ± 1.3 ^b^	0.000	5
Salty	3.1 ± 1.6	4.2 ± 1.0	4.0 ± 0.9	0.087	5
Spicy	4.0 ± 0.6 ^c^	5.2 ± 1.2 ^b^	7.1 ± 1.1 ^a^	0.000	5

The values in the rows marked with different letters differ statistically significantly at *p* ≤ 0.05.

## Data Availability

The data presented in this study are available on request from the corresponding author.

## Abbreviations

The following abbreviations are used in this manuscript:

FSL	flower shoot leaves
FSS	flower shoot stems
LSL	leafy shoot leaves
LSS	leafy shoot stems
